# Herbivory Dominates the Spring Diet of American Black Bears ( *Ursus americanus* ) in a Wood Bison ( *Bison bison athabascae* ) Neonatal Range, Suggesting Minimal Bison Consumption

**DOI:** 10.1002/ece3.72161

**Published:** 2025-09-15

**Authors:** Molly E. Sharp, Scott E. Nielsen, Mark A. Edwards

**Affiliations:** ^1^ Department of Renewable Resources University of Alberta Edmonton Alberta Canada; ^2^ Alberta Environment and Protected Areas, Government of Alberta Edmonton Alberta Canada

**Keywords:** black bear, diet analysis, predation, wood bison

## Abstract

Studying an organism's foraging behavior, especially for predator species, provides insight into their ecology, habitat needs, and interspecific relationships. American black bears (*Ursus americanus)* are generalist omnivores, with a diet primarily composed of vegetation and are known predators of a number of ungulate species, particularly their neonates. In this study, we analyzed the spring diet of black bears occupying the neonatal range of a small, threatened wood bison ( *Bison bison athabascae* ) herd in the Ronald Lake area of northeast Alberta to determine the predation risk of neonate bison. To estimate black bear consumption rates of bison, we used scat analysis and DNA metabarcoding to describe the spring diet of bears occupying the Ronald Lake wood bison herd's (RLBH) neonatal range. If black bears occupying the RLBH's neonatal range are consuming bison, either through predation or scavenging, then we would expect bison DNA to be present in black bear scats. We predicted that the increased availability of neonate bison in the spring would provide bears with greater predation and scavenging opportunities. Conversely, if black bear predation risk is low within the RLBH's neonatal range, then we would predict that herbaceous plants would dominate black bear diet early in the spring and berries later in the spring and summer. The spring diet of black bears was dominated by herbaceous and fruiting plants. Bison DNA, without visual evidence of animal remains, was found in only 1 of 79 scat samples (1.3%). Our results suggest that consumption rates of bison are low and that predation risk to neonate wood bison, during the RLBH's occupancy of their neonatal range, from black bears is likely minimal despite the two species' overlap in space and time.

## Graphical abstract

In this study, we used scat analysis and DNA metabarcoding to analyze the spring diet of black bears in the neonatal range of a small, threatened wood bison herd in northeast Alberta to assess predation risk of neonate bison. Our results suggest that consumption rates of bison are low and that predation risk to neonate wood bison, during the RLBH's occupancy of their neonatal range, from black bears is likely minimal.

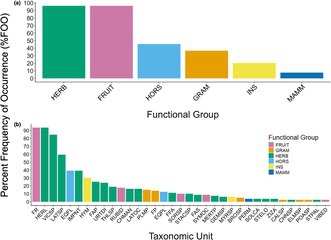

## 1 Introduction

Studying an organism's foraging decisions and feeding ecology can provide insights into its behavior, habitat requirements, and influence on other species in an ecosystem (Robbins 1983). For predators, dietary studies are essential to determine their role in an ecosystem and their influence on prey populations (Klare et al. 2011; Mills 1989). Predator species may be described as specialists or generalists based on their dietary preferences and foraging strategies (Andersson and Erlinge 1977; Futuyma and Moreno 1988; Wimp et al. 2019). Specialist predators have high fidelity to specific prey types and are well adapted for hunting a given prey species (Andersson and Erlinge 1977). In contrast, generalist predators display a more diverse diet, exploiting an array of resources based on availability (Wimp et al. 2019). Generalists respond to prey abundance by increasing search efforts during times of increased prey availability (pulses) or susceptibility (Huggler et al. 2023). Predator species respond to reproductive pulses of ungulates to exploit the vulnerability of neonates during the spring calving season (Barber‐Meyer et al. 2008; Huggler et al. 2023; McLaren et al. 2021; Rayl et al. 2018). Rayl et al. (2018) suggest that American black bears ( *Ursus americanus* ), a generalist species, shift their foraging tactics in response to the availability of neonate ungulates and will actively seek out areas where ungulate calves are most vulnerable. However, individual bears vary in predatory tendencies, with some individuals more actively searching out prey than others (Bastille‐Rousseau et al. 2011). Predation can serve as a significant source of neonatal mortality (Ballard 1992; Barber‐Meyer et al. 2008; Forrester and Wittmer 2019; Wolf et al. 2021), and bears have been identified as predators of neonate ungulates in predator–prey systems (Ballard 1992; Barber‐Meyer et al. 2008; Merkle et al. 2017; Popp et al. 2018; Wolf et al. 2021). For example, a study conducted by Moore et al. (2024) reported that 71% of moose calf mortalities (over a study period of 5 years) were caused by predation events, with the highest amount of predation events occurring within the first week of life. Black bear population density is suggested to influence predation risk to neonate ungulates; in systems with high black bear density, bears often account for a significant amount of neonatal mortality due to high encounter rates (Bastille‐Rousseau et al. 2011; Moore et al. 2024).

Black bears are described as generalist omnivores (McLaren et al. 2021) with a diet primarily composed of vegetation and supplemented with animal matter (Baldwin and Bender 2009; Mosnier et al. 2008; Popp et al. 2018; Raine and Kansas 1990). Resource availability strongly influences bear diets (Nielsen et al. 2017; Svoboda et al. 2019), which leads to high seasonal diet variability (Beeman and Pelton 1980; Mosnier et al. 2008; Raine and Kansas 1990). Due to their consumption of seasonally abundant food resources, black bear diets are often grouped into distinct seasons classified by diet composition (Beeman and Pelton 1980; Mosnier et al. 2008; Raine and Kansas 1990). Black bear spring diet (den emergence to late June) is characterized as having a high proportion of graminoid and herbaceous plant consumption (Beeman and Pelton 1980; Mosnier et al. 2008; Raine and Kansas 1990), as well as the highest rates of ungulate consumption (McLaren et al. 2021; Mosnier et al. 2008). In the late spring/early summer (mid‐June to late July) black bears have been found to consume a large proportion of insects, specifically ants and wasps (Raine and Kansas 1990). Once berries ripen in the summer (mid‐ to late July) black bear diet shifts to be dominated by berries, which have a high carbohydrate content (Rode and Robbins 2000), to obtain energy reserves for hibernation (Beeman and Pelton 1980; Raine and Kansas 1990; Welch et al. 1997).

This study explored whether black bears pose a predation risk on neonates from the Ronald Lake wood bison herd in northeast Alberta, Canada. Although wolves ( *Canis lupus* ) are more typical predators of bison (Carbyn and Trottier 1987; Shave et al. 2020), black bears have been identified as predators of other neonate ungulates and are the most important source of neonatal mortality for some elk ( *Cervus canadensis* ) and moose ( *Alces alces* ) populations (Bertram and Vivion 2002; McLaren et al. 2021; Merkle et al. 2017; Popp et al. 2018; Zager and Beecham 2006); therefore, exploration into the predator–prey relationship between black bears and wood bison is warranted. In the late spring of each year, the Ronald Lake bison herd (RLBH) migrates ~30 km to their neonatal range, consisting of an upland meadow complex and the surrounding upland forest along the base of the Birch Mountains (Belanger et al. 2017). Previous work with camera traps deployed in the RLBH's neonatal range showed that black bear activity in the meadow increases when bison and their calves are present in the spring, and wolf activity in the meadow is low (Belanger et al. 2017). Predator–prey dynamics between black bears and wood bison are unknown for the RLBH, and more generally, on bison. However, black bear predation on neonates may affect the population of this small herd.

In this study, we used DNA metabarcoding on black bear scat collected within the neonatal range of the RLBH to examine black bear spring diet and to assess predation risk on neonate wood bison. We sought to quantify the components of the spring diet for black bears that occupy the herd's neonatal range, where bison congregate with neonates after a short migration from their core range (Belanger et al. 2017; Hecker et al. 2025). We predicted that the increased availability of neonate bison in the spring would provide bears with greater predation and scavenging opportunities. If black bears occupying the RLBH's neonatal range are consuming bison, either through predation or scavenging, then we would expect bison DNA to be present in black bear scats. Conversely, if black bear predation risk is low within the RLBH's neonatal range, then we would predict that DNA from herbaceous plants would dominate black bear diet early in the spring and berries later in the spring. The presence or absence of bison in black bear diet will help inform future research into the relationship between black bears and bison within the RLBH's spring range and will determine if subsequent, more detailed predation studies are required.

## 2 Materials and Methods

### 2.1 Study System

The Ronald Lake wood bison herd is composed of ~270 individuals (ECCC 2018; AEP, personal communication, 2021) with a range that extends north of Fort McKay, west of the Athabasca River to the base of the Birch Mountains, and into the southern portion of Wood Buffalo National Park (Rawleigh et al. 2021). Wood bison of the Ronald Lake area are classified as Threatened under Alberta's Wildlife Act (Government of Alberta 2021). At the federal level, wood bison are listed as Threatened on Schedule 1 in the Species at Risk Act (SARA) (ECCC 2018).

**FIGURE 1:**
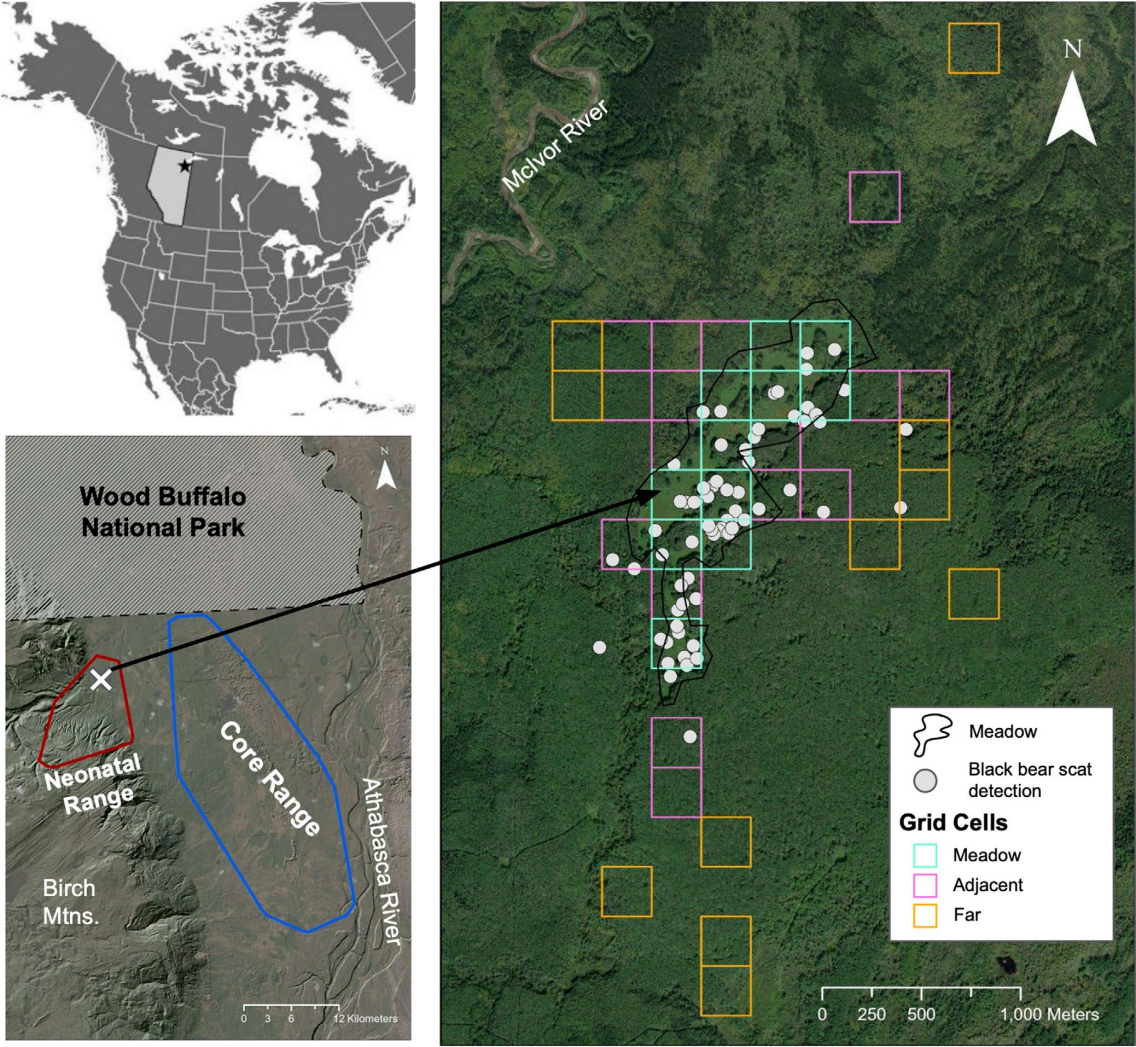
Aerial image of the study area and grid cells surveyed (*n* = 38) with the spatial distribution of black bear scats detected (*n* = 79) and used to describe the spring diet of black bears that occupy the Ronald Lake wood bison herd's neonatal range in northeastern Alberta, Canada. Grid cells were 250 m by 250 m in size (6.25 ha) and were categorized by their location relative to the open meadow (black outline). The inset map (top left) provides the location of the study area relative to North America. The aerial image at the bottom left displays minimum convex polygon home ranges for the core range (blue outline) and the neonatal range (red outline) of the RLBH with an “X” representing the location of the open meadow where bison congregate most. The open meadow is outlined in the aerial image by a black polygon.

The study area (Figure 1) is located in the Boreal Plains Ecozone (Parks Canada 2003) of northeast Alberta, Canada. The Boreal Plains Ecozone is characterized by short, warm summers (with a mean daily temperature in July ranging from 12.5°C to 17.5°C) and long, cold winters (with a mean daily temperature in January ranging from −17.5°C to −22.5°C) (Parks Canada 2003). The dominant tree species in upland deciduous forests in the area is trembling aspen ( *Populus tremuloides* ), and the dominant tree species in upland coniferous forests in the area include white spruce ( *Picea glauca* ) and jack pine (*Pinus banksia*) (Hecker et al. 2021). Common ungulate species of the study area include wood bison, white‐tailed deer ( *Odocoileus virginianus* ), and moose. Additionally, a variety of small mammalian prey species, such as snowshoe hare ( *Lepus americanus* ), voles (*Myodes* spp.), and deer mice ( *Peromyscus maniculatus* ), are common to the area. Black bear densities in northeastern Alberta are estimated to be approximately 333–370 bears per 1000 km^2^ (Alberta Environment and Parks 2016).

Common plants in the study area include wild red raspberry (*Rubus ideas*), saskatoon ( *Amelanchier alnifolia* ), red‐osier dogwood ( *Cornus alba* ), cow parsnip ( *Heracleum lanatum* ), and vetch (*Vicea* spp.). Many of these plant species are common spring and early summer diet items for black bears.

### 2.2 Field Methods

We surveyed for black bear scat in the neonatal range over 19 days in June and July of 2023, shortly after the bison left their spring range. A fishnet grid of 250 m by 250 m grid cells was superimposed over the Ronald Lake wood bison herd's spring range using ArcGIS Pro 3.2.0 Fishnet Function (Esri 2023). We categorized grid cells as “meadow” (cell is composed of at least 50% meadow), “adjacent” (cell is composed of less than 50% meadow and within a 500 m radius of the meadow), and “far” (500 m to 3 km away from meadow). A stratified random sampling technique was then used to select 90 grid cells for potential field surveys, with 38 grid cells (Figure 1) visited and surveyed, attempting to balance sampling effort in each stratum (12 meadow, 15 adjacent, and 11 far). A study by Güthlin et al. (2012) suggests that using transects for scat surveys is a reliable and unbiased method for collecting predator scat. Therefore, for our study, each grid cell was mapped to have East to West transects 20 m apart along the length of the grid cell. If a game trail was discovered, we followed the trail to the edge of the grid cell in all directions. We also used GPS tracks for all grid cell surveys and followed game trails to quantify search effort.

Visual reference guides (Chame 2003) were used to aid in the identification of black bear scats in the field. Black bear scats encountered along game trails and during transect surveys of the grid cells were collected, and the coordinates (NAD83 UTM Zone 12) were recorded. For each scat discovered, we noted the dimensions (length, width, and height), freshness (freshness scale modified from Gosselin et al. 2017), color, consistency, and odor (see Table S2 in Supporting Information). Approximately 30 mL of fecal matter was then collected from the middle of each scat to reduce surface contamination, and the sample was placed in a ziplock bag with silica gel beads (at a ratio of 4 mL silica gel beads to 1 mL scat) to desiccate the sample (Murphy et al. 2002).

### 2.3 Statistical Analysis

All statistical analyses were conducted in R v4.3.0 statistical software (R Core Team 2021). We examined the distributions of each parameter using histograms and boxplots. We used the Shapiro–Wilk test to assess normality. To compare the number of black bear scats found in meadow, adjacent, and far grid cells, we conducted a non‐parametric Kruskal–Wallis test. A post hoc Dunn test was conducted for pairwise comparisons using the R package “ggstatsplot” (Patil 2021).

### 2.4 DNA Metabarcoding

Black bear scat samples (10 mL of fecal material) were shipped to Jonah Ventures environmental DNA laboratory (5485 Conestoga Ct #210, Boulder, CO 80301) for an omnivore diet composition analysis using DNA metabarcoding. At Jonah Ventures laboratory, genomic DNA from black bear scat samples was extracted using the DNeasy 96 PowerSoil Pro Kit (384) (Qiagen, catalog number 47017). Genomic DNA was eluted into 100 μL and frozen at −20°C until the PCR was to be run. All sequencing was conducted using an Illumina MiSeq Sequencing System (5200 Illumina Way, San Diego, CA 92122). For plant DNA, a portion of the chloroplast *trnL* intron was PCR amplified from each genomic DNA sample using the c and h *trnL* primers (Craine et al. 2015; Taberlet et al. 2007). Plant DNA was PCR amplified under the following conditions: initial denaturation at 94°C for 3 min, followed by 40 cycles of 30 s at 94°C, 30 s at 55°C, 1 min at 72°C, and a final elongation at 72°C for 10 min. Vertebrate DNA was analyzed by a PCR amplification of the 12S rRNA gene (Ac12S primer) (hereafter we will refer to this PCR assay as 12SVert) (Evans et al. 2016). Vertebrate DNA was PCR amplified under the following conditions: initial denaturation at 94°C for 3 min, followed by 45 cycles of 30 s at 94°C, 30 s at 52°C, 1 min at 72°C, and a final elongation at 72°C for 10 min. For arthropod DNA, a portion of the mitochondrial cytochrome c oxidase subunit I (COI) gene was PCR amplified from each sample using the ZBJ‐ArtF1c and ArtR2c primers (hereafter we will refer to this PCR assay as ArthCOI) (Zeale et al. 2011). Arthropod DNA was PCR amplified under the following conditions: initial denaturation at 94°C for 5 min, followed by 45 cycles of 30 s at 94°C, 45 s at 45°C, 45 s at 72°C, and a final elongation at 72°C for 10 min.

A second round of barcoding PCR was performed for each DNA group (plant, vertebrate, and arthropod DNA) using template DNA (cleaned amplicon from the first PCR reaction) to complete the sequencing library construct. For the PCR amplification of plant DNA, an initial denaturation of 95°C for 3 min was followed by 8 cycles of 95°C for 30 s, 55°C for 30 s, and 72°C for 30 s. For the vertebrate barcoding PCR, an initial denaturation of 95°C for 3 min was followed by 8 cycles of 95°C for 30 s, 55°C for 30 s, and 72°C for 30 s. The arthropod barcoding PCR consisted of an initial denaturation of 95°C for 3 min followed by 8 cycles of 95°C for 30 s, 55°C for 30 s, and 72°C for 30 s.

For each sample, reads were clustered using the unoise3 denoising algorithm (Edgar 2016) as implemented in vsearch (Rognes et al. 2016) and raw sequences observed less than 8 times were discarded. Read counts of the resulting exact sequence variants (ESVs) were compiled. For each ESV, taxonomy was assigned using a custom best‐hits algorithm and a reference database consisting of publicly available sequences (GenBank; Benson et al. 2005), as well as Jonah Ventures voucher sequences records. Taxonomy was then generated using either all 100% matching reference sequences or all reference sequences within 1% of the top match, accepting the reference taxonomy for any taxonomic level with > 90% agreement across the top hits.

From the ESV data provided by Jonah Ventures, we then used the Basic Local Assignment Search Tool (BLAST) from the National Center for Biotechnology Information (NCBI 2018) to select the taxonomic units that had the highest percent match and that were known to occur within our study area as the taxonomic units for subsequent analyses (Hecker et al. 2021). If more than one species had the exact percent match for a gene sequence, we used the genus or family as the operational taxonomic unit (Hecker et al. 2021). We also organized taxonomic units into broader functional groups for an overall diet summary.

### 2.5 Diet Content Analysis

DNA metabarcoding reports the number of times a unique gene sequence is read within a sample. We calculated each taxonomic unit and broader functional group's relative read abundance (RRA). RRA was calculated as the read count of a particular taxonomic unit or functional group in a scat sample divided by the total number of reads across all taxonomic units for that specific sample, multiplied by 100 (Equation 1, Deagle et al. 2019; Hecker et al. 2021). This calculation was repeated for each scat sample, and we then calculated the mean RRA for each taxonomic unit and functional group to provide an overall diet summary (Deagle et al. 2019). Additionally, we calculated the percent frequency of occurrence (%FOO) for each taxonomic unit and functional group. The frequency of occurrence is the number of samples that contain a particular taxonomic unit (Deagle et al. 2019). We calculated the %FOO as the number of samples a distinct taxonomic unit or functional group was present in, divided by the total number of scat samples, multiplied by 100 (Equation 2, Deagle et al. 2019). In our final diet content estimates, we only included taxonomic units within our study area that accounted for at least 1% of the diet to address background sequencing errors (Deagle et al. 2019; Hecker et al. 2021).

(1) $${\mathrm{RRA}}_{i}=\frac{n_{i,k}}{R_{k}}\times 100\%$$

where *R*_*k*_ is the total number of reads in sample *k*, and *n*_*i,k*_ is the number of sequences for taxonomic unit *i* in sample *k*.

(2) $$\%{\mathrm{FOO}}_{i}=\frac{\sum_{k=1}^{S}l_{i,k}}{S}\times 100\%$$

where *S* is the number of samples and *l*_*i,k*_ is an indicator factor where *l*_*i,k*_ = 1 if the taxonomic unit *i* is present in sample *k*, and 0 if it is not.

## 3 Results

### 3.1 Black Bear Scat Surveys

**FIGURE 2:**
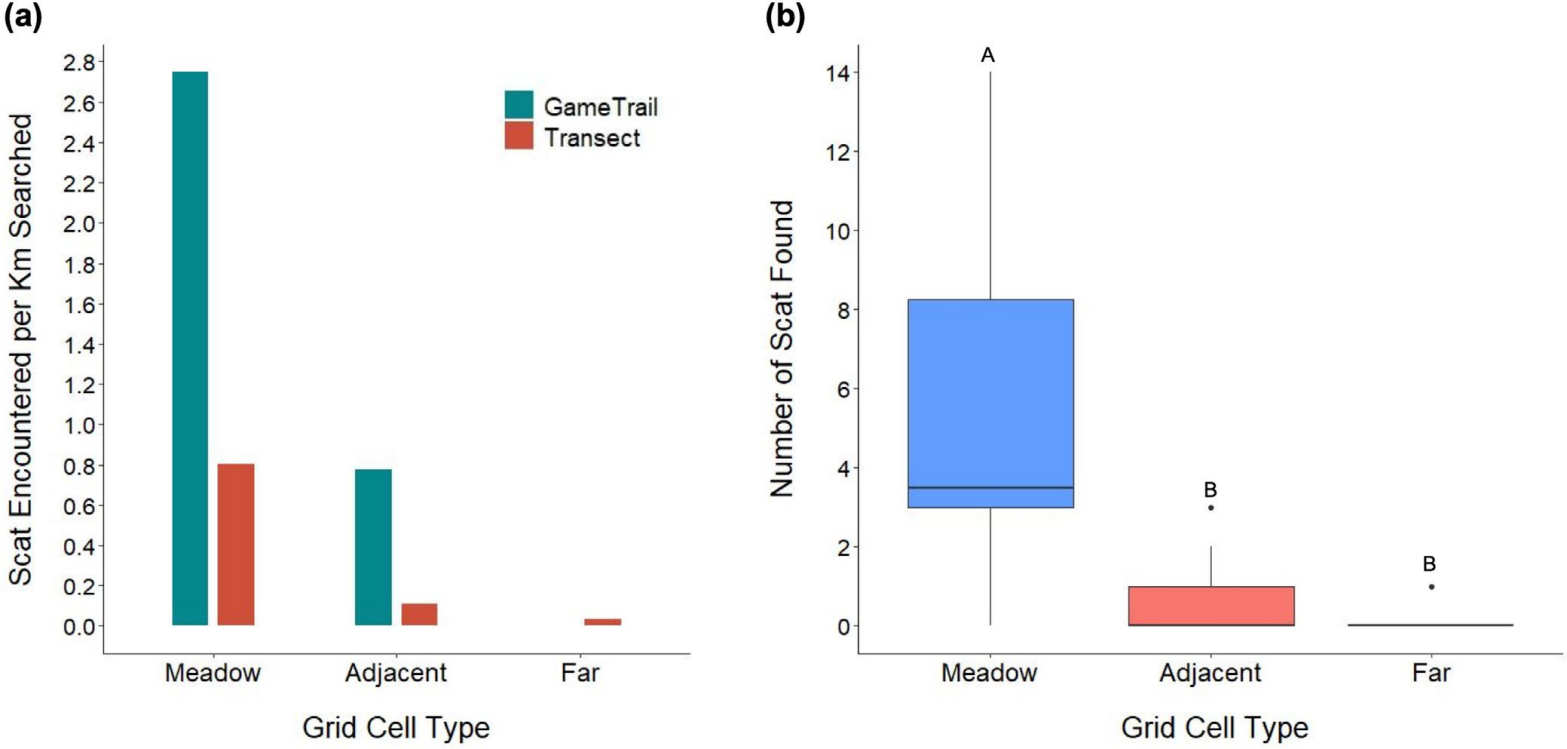
Collection rates of black bear scat (scats/km) in northeastern Alberta, Canada, based on grid cell type with respect to the open meadow where wood bison congregate in the spring and the type of survey (transect or game trail) (a). Total length of transects surveyed was 108.00 km, and the total length of game trails surveyed was 33.28 km. Number of black bear scat found for each grid cell location strata (b). The median for the number of scats found in meadow grid cells was 3.5 (IQR = 5.25). The median for the number of scats found in adjacent grid cells was 0.0 (IQR = 1.00), and the median for the number of scats found in far grid cells was 0.0 (IQR = 0.00).

We surveyed 141.28 km of transects and game trails over the 19 sampling days and collected 79 black bear scat samples. The overall collection rate was 0.56 scats/km, with the highest collection rates occurring in meadow grid cells during game trail (2.75 scats/km) and transect (0.81 scats/km) surveys (Figure 2a). For adjacent grid cells, the collection rate for game trail surveys was 0.78 scats/km, while transect surveys were 0.11 scats/km (Figure 2a). No black bear scat was found along the 2.29 km of game trails surveyed in far grid cells, and the collection rate for transect surveys in far grid cells was only 0.04 scats/km (Figure 2a). A significant difference was found between the number of scats encountered in each grid cell type (Figure 2b; Kruskal Wallis Test, *χ* ^2^ = 18.556, *p* = 0.017, df = 8). We found a significant difference between the number of black bear scats found in meadow and adjacent grid cells (post hoc Dunn test; *p* < 0.001) and the number of black bear scats found in meadow and far grid cells (post hoc Dunn test; *p* < 0.001). There was no significant difference between the number of black bear scats found in adjacent and far grid cells (post hoc Dunn test; *p* = 0.785).

### 3.2 Diet Composition

From the 79 collected black bear scat samples, 1,205,128 metabarcoding reads were generated from the *trnL* assay (plant DNA); for the 12SVert assay (vertebrate DNA) 96,461 metabarcoding reads were generated, and 866,672 metabarcoding reads were generated from the ArthCOI (invertebrate DNA) assay. The *trnL assay* identified 289 unique ESVs, from which we identified 48 taxonomic units (Table A1) based on genetic similarity and occurrence within the study area. The 12SVert assay (vertebrate DNA) identified 40 unique ESVs, which we reduced to 5 prey species (*Myodes* sp., *Myodes rutilus* , *Peromyscus maniculatus* , *Lepus americanus* , and *B. b. athabascae*). A majority of the reads (95,798 reads) from the 12SVert assay were attributed to black bear DNA and, therefore, were omitted from our analyses. Bison DNA was found in one scat sample collected in a meadow grid cell. The ArthCOI assay (invertebrate DNA) identified 1083 unique ESVs (see Table S1 in Supporting Information the number of DNA metabarcoing reads summarized by Order and Family). Many of these ESVs were associated with dung‐touching insects (such as the rove beetle *Ontholestes cingulatus*) that would have interacted with the black bear scat post‐excretion and, therefore, were not included in diet composition analyses. We only included reads for colonial insects from the order Hymenoptera in analyses, as insects from this order are most commonly reported in black bear diets (Baldwin and Bender 2009; Koike et al. 2012; McLaren et al. 2021; Proctor et al. 2023; Romain et al. 2013), with other insects potentially represented as those coming into contact with the scat post‐deposition. Twelve ESVs were assigned to the order Hymenoptera, and 5 of these ESVs were further reduced to the genus Myrmica.

**FIGURE 3:**
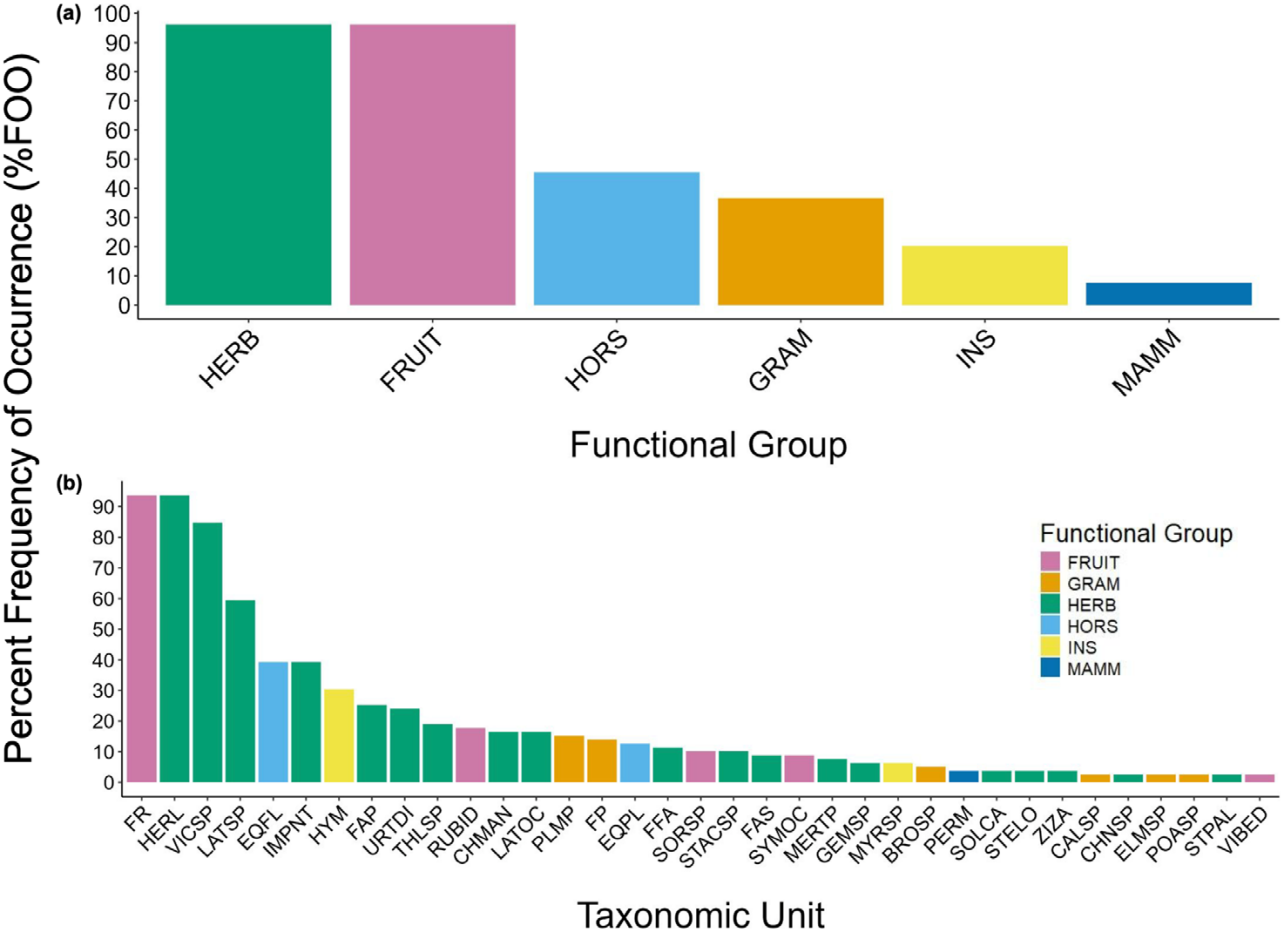
Spring diet composition of black bears ( *Ursus americanus* ) in northeastern Alberta, Canada, as estimated from percent frequency of occurrence (%FOO). %FOO was calculated as the number of samples a distinct taxonomic unit or functional group was present in, divided by the total number of scat samples, multiplied by 100. Gene sequences of dietary items were categorized into eight broad functional groups: herbaceous plants (HERB), fruiting plants (FRUIT), horsetails (HORS), insects (INS), graminoids (GRAM), and mammals (MAMM) (a) and then further reduced to more precise taxonomic units (b). Only taxonomic units present in more than one scat sample are displayed (see Table A1 in the Appendix for taxonomic units corresponding with codes shown on the *x*‐axis).

Herbaceous plants (HERB; flowering plants that do not produce fleshy fruits and woody stems) and fruiting plants (FRUIT; plants that produce fleshy fruits) both had a %FOO with a value of 96.21% (Figure 3a). Horsetails (HORS), graminoids (GRAM), and insects had a %FOO of 45.57%, 36.71%, and 20.25%, respectively. Mammals displayed the lowest %FOO of 7.59% (Figure 3a). When breaking up %FOO analyses into lower taxonomic units, family Rosacea (FR) and cow parsnip (*Heracleum lanatum*, HERL) displayed the highest %FOO of 93.67% (Figure 3b). Vetch species (*Vicea* spp., VICSP) displayed the second‐highest %FOO of 84.81% (Figure 3b). Peavine (*Lathyrus* spp., LATSP) had a %FOO of 59.49%. All other taxonomic units had a %FOO of less than 40% (Figure 3b). Bison DNA was only present in one black bear scat sample, with a %FOO of 1.27%. Due to the low %FOO of insects and mammals in comparison to plants, as well as digestion differences and varying recovery biases between taxa (De Barba et al. 2014), we focused our subsequent RRA analyses only on plants.

**FIGURE 4:**
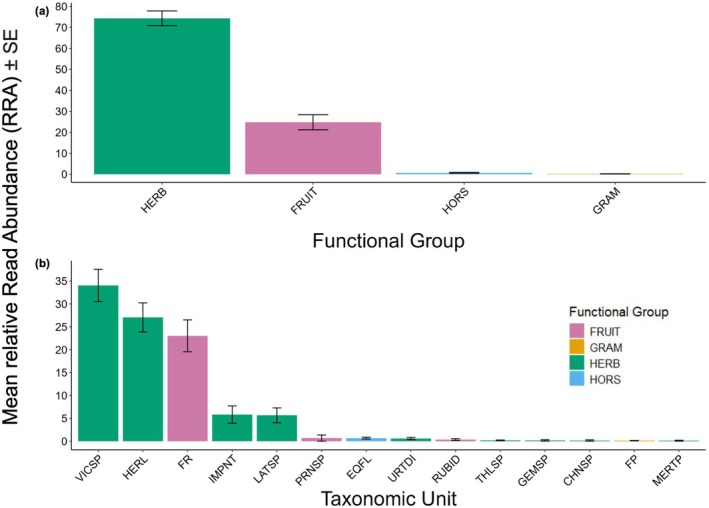
Plants consumed by black bears in the spring as estimated by mean relative read abundance (RRA). RRA was calculated as the read count of a particular functional group in a scat sample divided by the total number of reads across all taxonomic units for that specific sample, multiplied by 100. Error bars represent standard error. Gene sequences of plant dietary items were categorized into four broad functional groups: herbaceous plants (HERB), fruiting plants (FRUIT), graminoids (GRAM), and horsetails (HORS) (a) and then further reduced to more precise taxonomic units (b). (c) Only taxonomic units present in more than one scat sample are displayed (see Table A1 in the Appendix for taxonomic units corresponding with codes shown on the *x*‐axis).

Herbaceous plants (HERB) displayed the highest mean RRA (74.33 ± 3.52, hereafter all RRA values will be reported as mean ± standard error), and graminoids (GRAM) displayed the lowest mean RRA (0.22 ± 0.05) (Figure 4a). Fruiting plants (FRUIT) displayed the second‐highest mean RRA (24.76 ± 3.58) (Figure 4a). Mean RRA was 0.69 ± 0.20 for horsetails (HORS). When breaking up RRA analyses into lower taxonomic groups, vetch species (*Vicea* spp.; VICSP) have the highest mean RRA (34.07 ± 3.54), followed by cow parsnip ( *Heracleum lanatum* ; HERL) and family Rosaceae (FR) (26.07 ± 3.09 and 21.85 ± 3.37, respectively). All other plant taxonomic units had a mean RRA of less than 10 (Figure 4b).

## 4 Discussion

This study provides initial insights into the spring diet of black bears that overlap with the RLBH's neonatal range. Herbaceous and fruiting plants displayed both the highest %FOO and mean RRA, which supports our second prediction that black bear spring diet would be dominated by these two plant functional groups. Overall, our results highlight the diversity of black bear diets as opportunistic foragers. Our results suggest that black bear predation risk to neonate bison of the RLBH during spring occupancy of the meadow complex is likely minimal, as there was only one occurrence of bison DNA in the 79 (1.27 %FOO) collected black bear scat samples. This single occurrence of bison DNA could not be attributed to a predation or scavenging event, particularly since that scat did not show signs of bison consumption (no hair, bone, etc.) and could have resulted from environmental or laboratory contamination of the sample with bison DNA. Field evidence suggests that parturition occurs just prior to the RLBH's migration to the neonatal range, and thus, it is possible that neonates are only vulnerable to predation by black bears for the short window of time after parturition and before migration and the formation of a large nursery herd. The formation of a large nursery herd may serve as an anti‐predation tactic through increased vigilance and group defense (Carbyn and Trottier 1987).

The results of our diet analysis are consistent with previous research into black bear diet composition. Multiple black bear diet analyses have found the spring diet to be dominated by herbaceous plants (Beeman and Pelton 1980; McLaren et al. 2021; Romain et al. 2013), although we are not aware of any other study that overlapped with a bison neonatal range. For example, a study conducted by McLaren et al. (2021) on the spring diet of black bears in a moose and woodland caribou ( *Rangifer tarandus caribou* ) system found that the spring diet was dominated by herbaceous plants, with a %FOO of 97.3%. Moreover, McLaren et al. (2021) found that 27.5% of black bear scats contained animal matter, and when further analyzing consumed items by volume, they found that scats collected in Late May to early June contained the highest amounts of moose by volume. As herbaceous plants mature, crude fiber content increases, making plant material more difficult for black bears to digest (Furusaka et al. 2017). Therefore, in the late spring and summer, black bears consume higher amounts of fruiting plants because berries contain high amounts of carbohydrates in an easy‐to‐digest form (Rode and Robbins 2000). A study on the seasonal feeding ecology of black bears conducted by Beeman and Pelton (1980) highlighted seasonal differences in black bear diet. They found that in spring, black bear diet was composed of 90% grasses and herbaceous plants, whereas during summer and fall, it shifted primarily to fruits and seeds (Beeman and Pelton 1980). In our study, fruiting plants displayed the third‐highest mean RRA, and plants from the rose family (Rosacea) displayed the highest %FOO.

The open meadow within the RLBH's neonatal range is abundant in fruiting plant resources, such as wild red raspberry (Buitrago Gutierrez 2024). A study conducted by Buitrago Gutierrez (2024) suggests that the RLBH's spring range has higher shrub and forb biomass than other areas within the herd's range. Our results suggest that black bears favor the open meadow within the RLBH's spring range, as more scats were collected within meadow grid cells than adjacent and far cells (Figure 2). Specifically, we found 3.5 times more scat per km of game trails searched in meadow versus adjacent forest grid cells, and 7.2 times more scat were found per km of transect surveyed in meadow versus adjacent forest grid cells (Figure 2a). These results agree with previous studies, which have shown that bears select areas abundant in food resources, particularly fruit in mid‐to‐late summer (Davis et al. 2006; Nielsen et al. 2017; Svoboda et al. 2019). Specifically, a study conducted by Bastille‐Rousseau et al. (2011) found that black bears did not actively seek out areas with a high chance of encountering neonate ungulates; rather, they displayed a strong selection for areas abundant in vegetation. However, Bastille‐Rousseau et al. (2011) highlight that black bear selection for areas abundant in vegetation, partnered with frequent movement between patches, could increase their chance of encounter with neonate ungulates. Therefore, the most beneficial foraging strategy for black bears occupying the RLBH's neonatal range may be to actively seek out vegetation‐rich areas, such as the open meadow within the RLBH's spring range, and to capitalize on chance encounters with neonate bison while occupying a similar range (Bastille‐Rousseau et al. 2011).

The broad diet consumed by black bears may lead to niche overlap with herbivorous species, such as ungulates (Rioux et al. 2022). A study conducted by Rioux et al. (2022) explored trophic niche partitioning between black bears, moose, and caribou. The results of their study highlight significant trophic niche overlap between black bears and both ungulate species, and Rioux et al. (2022) suggest that this overlap may increase encounter rates and chance of predation. The locations of the black bear scats we collected (Figure 1) suggest a notable degree of spatial overlap between bears and bison within the RLBH's neonatal range, which could suggest trophic niche partitioning between these two species. Moreover, a study conducted by Buitrago Gutierrez (2024) described the spring diet of the RLBH and found that the most prevalent browse items bison consumed were wild prickly rose ( *Rosa acicularis* ) and wild raspberry ( *Rubus idaeus* ). Wild rose and raspberry both belong to Family Rosaceae, which was one of the most abundant taxonomic units found in our black bear diet analysis (93.67 %FOO, 26.07 ± 3.09 RRA). This dietary overlap observed between bison and black bears that occupy the RLBH's neonatal range could indicate competition for food resources, which warrants future study into the relationship between these two species.

Though black bears consume a wide variety of plants, their digestive physiology is better suited to break down animal matter (Bowersock et al. 2021). Differences in digestibility between food items have the potential to influence scat analysis results, as food items that display a higher digestibility will result (e.g., animal matter) in less fecal remains (both physical and molecular) (Elfström et al. 2013). Food items with lower digestibility, such as plant matter, have a shorter gut retention time and will result in more fecal remains (Elfström et al. 2013). Given this, the high %FOO and RRA for plant items in our study could be, in part, due to a high amount of plant remnants present in scat samples. Though there is an identified bias in the presence of fecal remains between food items, DNA metabarcoding of fecal material is suggested to provide a credible estimate of population‐level diets (Deagle et al. 2019) and molecular analyses of scat reflect a non‐invasive sampling technique that can be implemented in many study systems. Another consideration when using fecal matter for molecular diet analysis is the preservation of DNA in scat samples. DNA degradation due to the digestion process and exposure to harsh environmental conditions after scat is deposited could influence the success of DNA metabarcoding; however, a study conducted by Massey et al. (2021) suggests that there is not a significant difference in the number of sequence reads between fresh and degraded scat samples. Regardless of these potential biases and shortcomings of DNA metabarcoding, Mumma et al. (2016) suggest that molecular diet methods had higher rates of detection than traditional morphological analysis, and therefore, we believe our use of DNA metabarcoding to summarize black bear spring diet is justified.

Although the results of our diet analysis revealed low amounts of bison DNA in black bear scats, black bears do select the open meadow, which is abundant in both plant food resources and neonate bison in the spring. One caveat is that black bear scat surveys were conducted just after the RLBH had exited their spring range due to animal care requirements of avoiding impacting behavior and activity of bison during sampling; therefore, it is possible that the scat samples were a combination of samples from the period when bison occupied the range to shortly after they left while we were conducting surveys. Future studies should explore the spatiotemporal context of black bears and bison within the meadow complex to provide additional insight into the interspecific relationship between the two species. This could be achieved by additional work with camera traps to develop multi‐species occupancy models (Feldman et al. 2024) or through a black bear radio‐collaring project to obtain better insight into black bear movement, habitat use, and individual variation (Nielsen et al. 2002). Additionally, a study focused on quantifying birth rates and neonate survival, as well as identifying causes of neonatal mortality, would provide insights on predation levels and neonate recruitment. This study provided insight into the spring diet for black bears that occupy the RLBH's neonatal range, and the results of our diet analysis suggest that wood bison make up a small proportion of black bear spring diet. Though black bear consumption rates of wood bison are suggested to be low, black bears do appear to select the open meadow within the Ronald Lake bison herd's neonatal range. Our study revealed spatial and dietary overlap between black bears and bison within the RLBH's neonatal range, which warrants future study into the interspecific relationship between bison and black bears.

## Author Contributions

**Molly E. Sharp:** conceptualization (lead), data curation (lead), formal analysis (lead), funding acquisition (supporting), investigation (lead), methodology (lead), project administration (equal), writing – original draft (lead), writing – review and editing (lead). **Scott E. Nielsen:** conceptualization (supporting), formal analysis (supporting), funding acquisition (lead), investigation (supporting), methodology (supporting), project administration (lead), resources (lead), software (lead), supervision (equal), validation (equal), visualization (supporting), writing – review and editing (supporting). **Mark A. Edwards:** conceptualization (supporting), funding acquisition (lead), investigation (supporting), methodology (supporting), project administration (lead), supervision (equal), validation (equal), visualization (supporting), writing – review and editing (supporting).

## Conflicts of Interest

The authors declare no conflicts of interest.

## Supporting information


**Appendix S1:** ece372161‐sup‐0001‐AppendixS1.docx.

## Data Availability

Data files with the number of DNA metabarcoding reads for each taxonomic unit, data used for statistical analysis, and R script has been uploaded to Dryad and can be accessed using this link: https://doi.org/10.5061/dryad.cc2fqz6hh.

## Appendix A

**TABLE A1:** Taxonomic unit codes.

Order	Family	Genus	Species	Taxonomic unit	TaxCode	Common
Alismatales	Araceae	Calla	*Calla palustris*	*Calla palustris*	CALPA	*Calla palustris*
Apiales	Apiaceae	—	—	Family Apiaceae	FAP	Carrots
Apiales	Apiaceae	Heracleum	*Heracleum lanatum*	*Heracleum lanatum*	HERL	Cow parsnip
Apiales	Apiaceae	Zizia	*Zizia aptera*	*Zizia aptera*	ZIZA	Heart‐leaved alexanders
Artiodactyla	Bovidae	Bison	*Bison bison athabascae*	*Bison bison athabascae*	BBA	Wood bison
Asterales	Asteraceae	Achilea	*Achilea millefolium*	*Achilea millefolium*	ACHM	Common yarrow
Asterales	Asteraceae	—	—	Family Asteraceae	FAS	Asters
Asterales	Asteraceae	Solidago	*Solidago canadensis*	*Solidago canadensis*	SOLCA	Goldenrod
Boraginales	Boraginaceae	Mertensia	*Mertensia paniculata*	*Mertensia paniculata*	MERTP	Bluebells
Brassicales	Brassicaceae	Arabis	—	*Arabis* spp.	ARSP	Cress (Mustards)
Caryophyllales	Amaranthaceae	Chenopodium	*Chenopodium album*	*Chenopodium album*	CHENA	Lamb's quarters
Caryophyllales	Amaranthaceae	Chenopodium	—	*Chenopodium* spp.	CHNSP	Lamb's quarters/strawberry blight
Caryophyllales	Caryophyllaceae	Stellaria	*Stellaria longifolia*	*Stellaria longifolia*	STELF	Long‐leaved chick weed
Caryophyllales	Caryophyllaceae	Stellaria	*Stellaria longipes*	*Stellaria longipes*	STELO	Long‐leaved starwort
Cornales	Cornaceae	Cornus	—	*Cornus* spp.	CORSP	Red‐osier dogwood/bunchberry
Dipsacales	Caprifoliaceae	Symphoricarpos	*Symphoricarpos occidentalis*	*Symphoricarpos occidentalis*	SYMOC	Western snowberry
Dipsacales	Adoxaceae	Viburnum	*Viburnum edule*	*Viburnum edule*	VIBED	Low bush‐cranberry
Equisetales	Equisetaceae	Equisetum	*Equisetum fluviatile*	*Equisetum fluviatile*	EQFL	Horsetail
Equisetales	Equisetaceae	Equisetum	*Equisetum palustre*	*Equisetum palustre*	EQPL	Horsetail
Equisetales	Equisetaceae	Equisetum	—	*Equisetum* spp.	EQSP	Horsetail
Equisetales	Equisetaceae	Equisetum	*Equisetum sylvaticum*	*Equisetum sylvaticum*	EQSY	Horsetail
Ericales	Balsaminaceae	Impatiens	*Impatiens noli‐tangere*	*Impatiens noli‐tangere*	IMPNT	Spotted touch‐me‐not
Fabales	Fabaceae	—	—	Family Fabaceae	FFA	Peas
Fabales	Fabaceae	Lathyrus	*Lathyrus ochroleucus*	*Lathyrus ochroleucus*	LATOC	Cream‐colored vetchling
Fabales	Fabaceae	Lathyrus	—	*Lathyrus* spp.	LATSP	Peavine
Fabales	Fabaceae	Vicia	—	*Vicia* spp.	VICSP	Vetch
Hymenoptera	—	—	—	Order Hymenoptera	HYM	Hymenoptera
Hymenoptera	Formicidae	Myrmica	—	*Myrmica* spp.	MYRSP	Ants
Lagomorpha	Leporidae	Lepus	*Lepus americanus*	*Lepus americanus*	LEPA	Snowshoe hare
Lamiales	Plantaginaceae	Plantago	*Plantago major*	*Plantago major*	PLTMA	Common plantain
Lamiales	Lamiaceae	Stachys	—	*Stachys* spp.	STACSP	Nettle
Lamiales	Lamiaceae	Stachys	*Stachys palustris*	*Stachys palustris*	STPAL	Swamp hedge‐nettle
Myrtales	Onagraceae	Chamaenerion	*Chamaenerion angustifolium*	*Chamaenerion angustifolium*	CHMAN	Fireweed
Poales	Poaceae	Bromus	—	*Bromus* spp.	BROSP	Brome/Grass
Poales	Poaceae	Calamagrostis	—	*Calamagrostis* spp.	CALSP	Grass
Poales	Poaceae	Elymus	—	*Elymus* spp.	ELMSP	Grass
Poales	Poaceae	—	—	Family Poaceae	FP	Grasses
Poales	Poaceae	Poa	—	*Poa* spp.	POASP	Grass
Proteales	Elaeagnaceae	Shepherdia	*Shepherdia canadensis*	*Shepherdia canadensis*	SHECA	Buffaloberry
Ranunculales	Ranunculaceae	Thalictrum	—	*Thalictrum* spp.	THLSP	Rue
Rodentia	Cricetidae	Myodes	*Myodes rutilus*	*Myodes rutilus*	MYRU	Red‐backed vole
Rodentia	Cricetidae	Myodes	—	*Myodes* sp.	MYSP	Vole
Rodentia	Cricetidae	Peromyscus	*Peromyscus maniculatus*	*Peromyscus maniculatus*	PERM	Deer mouse
Rosales	Rosaceae	—	—	Family Rosaceae	FR	Roses
Rosales	Rosaceae	Geum	*Geum macrophyllum*	*Geum macrophyllum*	GEMA	Yellow avens
Rosales	Rosaceae	Geum	—	*Geum* spp.	GEMSP	Avens
Rosales	Rosaceae	Prunus	—	*Prunus* spp.	PRNSP	Cherry
Rosales	Rosaceae	Rubus	*Rubus idaeus*	*Rubus idaeus*	RUBID	Wild red raspberry
Rosales	Rosaceae	Sorbus	—	*Sorbus* spp.	SORSP	Mountain ash
Rosales	Urticaceae	Urtica	*Urtica dioica*	*Urtica dioica*	URTDI	Stinging nettle
Rubiales	Rubiaceae	Galium	*Galium boreale*	*Galium boreale*	GALBO	Northern bedstraw
Sapindales	Sapindaceae	Acer	—	*Acer* spp.	ACSP	Maple
