# Supplementary material for: Herbivory Dominates the Spring Diet of American Black Bears ( Ursus americanus ) in a Wood Bison ( Bison bison athabascae ) Neonatal Range, Suggesting Minimal Bison Consumption
Source: Ecol Evol. 2025 Sep 15;15(9):e72161. doi: 10.1002/ece3.72161 (PMC12435978; doi:10.1002/ece3.72161)
Supplement: Supplementary file 1 — Appendix S1: ece372161‐sup‐0001‐AppendixS1.docx. [file ECE3-15-e72161-s001.docx]

## Supplementary Materials – Summary of Invertebrate DNA Metabarcoding Data

**Table S1.** Number of DNA metabarcoding reads from ArthCOI assay summarized by Order and Family. A dash (-) indicates that the reads were not identified to the family level.

| **Order** | **Family** | **Total Number of Reads** |
| --- | --- | --- |
| Adinetida | Adinetidae | 1112 |
| Araneae | Clubionidae | 613 |
| Araneae | Philodromidae | 31 |
| Araneae | Salticidae | 186 |
| Chromulinales | Chromulinaceae | 114 |
| Coleoptera | - | 345 |
| Coleoptera | Byturidae | 2566 |
| Coleoptera | Chrysomelidae | 67 |
| Coleoptera | Cryptophagidae | 10249 |
| Coleoptera | Curculionidae | 403 |
| Coleoptera | Hydrophilidae | 1419 |
| Coleoptera | Latridiidae | 65 |
| Coleoptera | Ptiliidae | 1010 |
| Coleoptera | Staphylinidae | 60934 |
| Diptera | - | 507 |
| Diptera | Anisopodidae | 133 |
| Diptera | Anthomyzidae | 14 |
| Diptera | Calliphoridae | 34 |
| Diptera | Carnidae | 955 |
| Diptera | Cecidomyiidae | 15672 |
| Diptera | Ceratopogonidae | 796 |
| Diptera | Chironomidae | 7300 |
| Diptera | Chloropidae | 84 |
| Diptera | Drosophilidae | 32316 |
| Diptera | Fanniidae | 975 |
| Diptera | Muscidae | 330865 |
| Diptera | Phoridae | 653 |
| Diptera | Psychodidae | 31641 |
| Diptera | Sciaridae | 198 |
| Diptera | Sepsidae | 11619 |
| Diptera | Simuliidae | 56 |
| Diptera | Sphaeroceridae | 17321 |
| Diptera | Stratiomyidae | 115087 |
| Diptera | Tabanidae | 373 |
| Entomobryomorpha | Entomobryidae | 32 |
| Hemiptera | - | 652 |
| Hemiptera | Aphididae | 38 |
| Hemiptera | Aphrophoridae | 172 |
| Hemiptera | Berytidae | 9 |
| Hemiptera | Cicadellidae | 66 |
| Hymenoptera | - | 6216 |
| Hymenoptera | Braconidae | 13 |
| Hymenoptera | Formicidae | 1321 |
| Hymenoptera | Tenthredinidae | 9 |
| Lepidoptera | Epermeniidae | 8 |
| Lepidoptera | Erebidae | 2590 |
| Lepidoptera | Geometridae | 49 |
| Lepidoptera | Nepticulidae | 47 |
| Lepidoptera | Noctuidae | 592 |
| Lepidoptera | Tortricidae | 76 |
| Mesostigmata | - | 84 |
| Psocoptera | - | 33 |
| Psocoptera | Caeciliusidae | 9015 |
| Psocoptera | Peripsocidae | 225 |
| Psocoptera | Psocidae | 18 |
| Rhabditida | - | 4011 |
| Rhabditida | Ascarididae | 3310 |
| Rhabditida | Panagrolaimidae | 310 |
| Rhabditida | Rhabdiasidae | 5020 |
| Rhabditida | Rhabditidae | 2240 |
| Rhabditida | Steinernematidae | 319 |
| Sarcoptiformes | Ceratoppiidae | 76 |
| Oribata | Ceratozetidae | 2273 |
| Endeostigmata | Nanorchestidae | 8 |
| Strongylida | Strongylidae | 8418 |
| Thysanoptera | - | 16 |
| Thysanoptera | Thripidae | 16949 |
| Trombidiformes | - | 25388 |
| Trombidiformes | Anystidae | 99 |
| Trombidiformes | Ereynetidae | 1080 |
| Trombidiformes | Eriophyidae | 38643 |
| Trombidiformes | Eupodidae | 596 |
| Trombidiformes | Tarsonemidae | 11 |
| Trombidiformes | Triophtydeidae | 26 |
| Unknown Order | - | 51416 |
|  | **Total Number of Reads** | 866223 |

**Supplementary Materials - Black Bear Scat Characteristics**

**Table S2.** Characteristics of black bear scats that were identified and collected in the field. A fishnet grid of 250 m by 250 m grid cells was superimposed over the RLBH’s spring range, with each grid cell assigned a unique column ID (letter) and row ID (number). Scat sample identification is a combination of the grid cell the scat was found in (e.g., S23, where “S” is the column ID and “23” is the row ID) and the number of scats found in a grid cell (e.g., S1, S2, S3, etc.).

| **Scat Sample** | **Length (cm)** | **Width (cm)** | **Height (cm)** | **Colour** | **Consistency** | **Odour** | **Freshness**  **Score^1^** |
| --- | --- | --- | --- | --- | --- | --- | --- |
| O19-S1 | 44 | 38 | 3 | Black | Crumbled | None | 3 |
| P15-S1 | 30 | 19 | 2 | Dark-brown | Dried | None | 3 |
| P17-S1 | 20 | 20 | 12 | Black | Well-formed | Slight | 2 |
| P17-S2 | 42 | 30 | 4 | Black | Formed | None | 4 |
| P17-S3 | 42 | 22 | 5 | Black | Loose | Slight | 4 |
| P17-S4 | 16 | 13 | 3 | Black | Soft | Slight | 4 |
| P17-S5 | 27 | 15 | 6 | Black | Well-formed | Slight | 2 |
| P17-S6 | 20 | 18 | 6 | Black | Loose | Slight | 2 |
| P17-S7 | 30 | 20 | 10 | Black | Loose | Slight | 2 |
| P17-S8 | 26 | 20 | 6 | Black | Well-formed | Slight | 2 |
| P17-S9 | 20 | 15 | 7 | Black | Well-formed | Slight | 2 |
| P17-S10 | 30 | 19 | 10 | Black | Well-formed | Slight | 2 |
| P17-S11 | 30 | 25 | 5 | Dark-brown | Well-formed | None | 3 |
| P17-S12 | 29 | 21 | 4 | Dark-brown | Crumbled | None | 4 |
| P17-S13 | 29 | 21 | 4 | Dark brown | Formed, dried | None | 4 |
| P18-S1 | 50 | 30 | 7 | Black | Loose | Slight | 3 |
| P18-S2 | 38 | 28 | 6 | Brown | Loose | None | 4 |
| P18-S3 | 38 | 30 | 5 | Brown | Very dried | None | 4 |
| P18-S4 | 25 | 19 | 5 | Black | Loose | Slight | 3 |
| P18-S5 | 25 | 23 | 3 | Brown | Formed, dried | None | 4 |
| P18-S6 | 32 | 20 | 9 | Black | Well-formed | Slight | 3 |
| P18-S7 | 18 | 15 | 5 | Black | Soft | Slight | 3 |
| P19-S1 | 20 | 16 | 6 | Black | Well-formed | Slight | 2 |
| P19-S2 | 40 | 23 | 5 | Black | Loose | None | 3 |
| P19-S3 | 26 | 15 | 6 | Black | Crumbled | None | 3 |
| P20-S1 | 35 | 35 | 10 | Black | Well-formed | Slight | 2 |
| P20-S2 | 30 | 20 | 4 | Black | Well-formed | Slight | 3 |
| P20-S3 | 32 | 16 | 12 | Black | Well-formed | Slight | 1 |
| P20-S4 | 55 | 27 | 4 | Black | Crumbled | None | 3 |
| P20-S5 | 30 | 27 | 7 | Dark brown | Crumbled | None | 3 |
| Q19-S1 | 23 | 16 | 6 | Black | Well-formed | None | 3 |
| Q19-S2 | 30 | 19 | 6 | White | Loose | None | 3 |
| Q19-S3 | 26 | 19 | 6 | Black | Loose | Slight | 3 |
| Q19-S4 | 24 | 16 | 5 | Black | Formed, dried | None | 3 |
| Q19-S5 | 36 | 32 | 8 | Dark brown | Loose | None | 3 |
| Q19-S6 | 24 | 24 | 3 | Dark brown | Crumbled | None | 2 |
| Q19-S7 | 22 | 22 | 2 | Dark brown | Loose | None | 2 |
| Q19-S8 | 26 | 18 | 5 | Dark brown | Loose | Slight | 3 |
| Q19-S9 | 32 | 24 | 9 | Black | Crumbled | None | 3 |
| Q19-S10 | 18 | 13 | 6 | Black | Crumbled | None | 3 |
| Q19-S11 | 50 | 12 | 8 | Black | Crumbled | None | 4 |
| Q19-S12 | 30 | 23 | 5 | Black | Well-formed | None | 2 |
| Q19-S13 | 25 | 17 | 15 | Black | Loose | None | 4 |
| Q19-S14 | 30 | 19 | 5 | Black | Loose | None | 3 |
| Q20-S1 | 38 | 29 | 4 | Black | Crumbled | None | 4 |
| Q20-S2 | 19 | 18 | 5 | Black | Crumbled | Slight | 3 |
| Q20-S3 | 36 | 18 | 5 | Brown | Well-formed | Slight | 3 |
| Q20-S4 | 25 | 20 | 9 | Brown | Formed | None | 3 |
| Q20-S5 | 47 | 20 | 7 | Brown | Well-formed | None | 3 |
| Q20-S6 | 34 | 22 | 9 | Green-brown | Well-formed | None | 4 |
| Q20-S7 | 16 | 14 | 10 | Black | Well-formed | Slight | 2 |
| Q20-S8 | 30 | 21 | 5 | Dark brown | Loose | None | 4 |
| Q20-S9 | 42 | 27 | 3 | Dark brown | Loose | None | 3 |
| Q20-S10 | 33 | 14 | 10 | Black | Well-formed | Slight | 2 |
| Q20-S11 | 44 | 30 | 9 | Black | Well-formed | Slight | 2 |
| Q20-S12 | 25 | 20 | 7 | Black | Well-formed | Slight | 3 |
| Q21-S1 | 30 | 20 | 5 | Brown | Crumbled | Slight | 4 |
| Q21-S2 | 21 | 16 | 7 | Black | Loose | Slight | 3 |
| Q21-S3 | NA | NA | NA | NA | NA | NA | NA |
| Q21-S4 | 40 | 30 | 4 | Black | Crumbled | None | 3 |
| Q22-S1 | 24 | 21 | 10 | Brown | Well-formed | None | 4 |
| Q22-S2 | 30 | 20 | 9 | Black | Formed | Slight | 1 |
| R20-S1 | 18 | 18 | 4 | Green-brown | Well-formed | Slight | 3 |
| R20-S2 | 40 | 25 | 10 | Black | Crumbled | None | 3 |
| R21-S1 | 33 | 24 | 4 | Black | Loose | None | 3 |
| R21-S2 | 30 | 12 | 5 | Black | Loose | None | 3 |
| R21-S3 | 16 | 14 | 6 | Black | Loose | None | 3 |
| R22-S1 | 26 | 24 | 7 | Black | Well-formed | Slight | 2 |
| R22-S2 | 32 | 22 | 4 | Black | Loose | Slight | 2 |
| R22-S3 | 40 | 15 | 10 | Black | Loose | Slight | 3 |
| S20-S1 | 30 | 20 | 3 | Black | Crumbled | None | 4 |
| S22-S1 | 30 | 26 | 7 | Black | Well-formed | Slight | 2 |
| S22-S2 | 27 | 19 | 8 | Black | Loose | Slight | 2 |
| S22-S3 | 50 | 8 | 3 | Dark brown | Loose | Slight | 4 |
| S23-S1 | 27 | 17 | 8 | Dark brown | Well-formed | Slight | 2 |
| S23-S2 | 24 | 20 | 7 | Black | Well-formed | Slight | 2 |
| S23-S3 | 35 | 27 | 2 | Brown | Loose | None | 4 |
| T22-S1 | 35 | 15 | 10 | Black | Well-formed | Slight | 1 |
| U20-S1 | 30 | 17 | 3 | Black | Loose | None | 4 |

^1^Freshness scale modified from Gosselin et al. (2017), where 1 = very fresh (i.e., deposited that day) and 4 = very old (i.e., many days old).
